# Use of urinary naloxone levels in a single provider practice: a case study

**DOI:** 10.1186/s13722-020-0178-9

**Published:** 2020-01-15

**Authors:** Jill S. Warrington, Kaitlyn Booth, Gregory S. Warrington, Samuel Francis-Fath

**Affiliations:** 1 Aspenti Health, South Burlington, VT USA; 2 Robert Larner School of Medicine, Department of Pathology and Laboratory Medicine, University of Vermont Medical Center, 111 Colchester Avenue, Burlington, VT 5401 USA; 3 Mathematics and Statistics, University of Vermont, Burlington, VT USA

**Keywords:** Naloxone, Urine drug testing, Spiking, Buprenorphine

## Abstract

**Background:**

Urine drug monitoring for medications for opioid use disorder (MOUD) such as buprenorphine can help to support treatment adherence. The practice of introducing unconsumed medication directly into urine (known as “spiking” samples) has been increasingly recognized as a potential means to simulate treatment adherence. In the laboratory, examination of the ratios of buprenorphine and its metabolite, norbuprenorphine, has been identified as a mechanism to identify “spiked” samples. Urine levels of naloxone may also be a novel marker in cases where the combination buprenorphine–naloxone product has been administered. This case study, which encompasses one provider’s practice spanning two sites, represents a preliminary report on the utility of using urinary naloxone as an indicator of “spiked” urine toxicology samples. Though only a case study, this represents the largest published evaluation of patients’ naloxone levels to date.

**Case presentation:**

Over a 3-month period across two practice sites, we identified 1,223 patient samples with recorded naloxone levels, spanning a range of 0 to 12,161 ng/ml. The average naloxone level was 633.65 ng/ml with the majority (54%) of samples < 300 ng/ml. 8.0% of samples demonstrated extreme values of naloxone (> 2000 ng/ml). One practice site, which had increased evidence of specimen tampering at collections, had a greater percent of extreme naloxone levels (>  2000 ng/ml) at 9.3% and higher average naloxone level (686.8 ng/ml), in contrast to a second site (570.9 ng/ml; 6.4% at > 2000 ng/ml) that did not have known reports of specimen tampering.

**Conclusions:**

We postulate that naloxone may serve as an additional flag to identify patient “spiking” of urine samples with use of the combination product of buprenorphine–naloxone.

## Background

Medications for opioid use disorder (MOUD) are critical tools for reducing opioid use, mitigating opioid withdrawal, enhancing treatment retention, as well as reducing the incidence of opioid-related overdoses, the acquisition of HIV and chronic hepatitis, and death among patients with opioid use disorder (OUD; [1, 2]). Urine drug testing has been advocated as an effective strategy to evaluate treatment adherence to MOUD drugs such as buprenorphine and methadone as well as to identify unexpected drug use in patients with substance use disorders [3].

Consideration for urine specimen tampering has been long advocated as an important part of urine drug testing [4]. Overall rates of urine specimen tampering in various urine drug testing settings, as determined by specimen validity testing, can range from approximately 1 to 3% of cases [5]. While urine specimen tampering is not an uncommon practice, there has been far less evaluation of how often patients “spike” urine with prescription medications, by inserting unconsumed drug directly into urine, to simulate treatment adherence [6]. Patients with OUD may be motivated to simulate treatment adherence for a variety of reasons including fear of repercussions if they aren’t adherent to medications, ability to divert/sell controlled substances, and/or desire to not disappoint their provider [7].

In the context of buprenorphine treatment, recent studies have identified the use of the ratio of buprenorphine and its metabolite, norbuprenorphine, to identify the practice of spiking buprenorphine products into a urine specimen [6, 8, 9]. Rates of unexpected norbuprenorphine:buprenorphine (N:B) ratios, as a marker of possible specimen adulteration with the prescribed medication, ranged from 0.6 to 27% of samples [10].

A limited number of reports have begun to examine the utility of naloxone as a marker of spiking the combination buprenorphine–naloxone product into urine specimens [11]. For example, Sobolesky et al. [12] identified the pattern of high buprenorphine, low norbuprenorphine and high naloxone in two patients, leading the authors to suspect these were “spiked” specimens to simulate treatment adherence.

While it is increasingly appreciated that naloxone levels are detectable in urine after sublingual administration of the combination buprenorphine–naloxone product [13], there is little characterization of what might constitute an elevated level of naloxone for the identification of potential specimen spiking with original drug into urine.

In this case study, we examined the naloxone levels in patients of one provider across two practice settings. Since one of the two sites had repeated anecdotal reports of specimen tampering at the point of urine collection, we were interested in whether differences in naloxone levels would be found across the two settings.

## Case presentation

Urine samples were collected by observed collection from June to August 2018 across two practice sites. During this time, we identified 1223 patient samples, spanning 275 unique patients, with recorded naloxone levels. Naloxone levels ranged from 0 to 12,161 ng/ml with an average naloxone level of 633.65 ng/ml (1 s.d. 1039.38). The median naloxone level was 253 ng/ml with the first quartile at 57.5 ng/ml and the third quartile at 762 ng/ml. 54% of samples were < 300 ng/ml. 8.0% of samples demonstrated extreme values of naloxone (> 2000 ng/ml).

Anecdotal reports from collectors performing observed collections suggested a relatively higher rate of attempts to tamper specimens at site 1 in contrast to site 2, despite shared staffing and common provider practice protocols between the two sites. Among the witnessed events, collectors identified theft of empty cups and “dumpster diving” for disposed urine cups behind the provider office. In addition, a review of specimen validity testing (creatine, specific gravity, and general oxidants) performed at both sites demonstrated a higher incidence of abnormal specimen validities at site 1 (30 patient samples) in comparison to site 2 (18 patient samples).

**Fig. 1:**
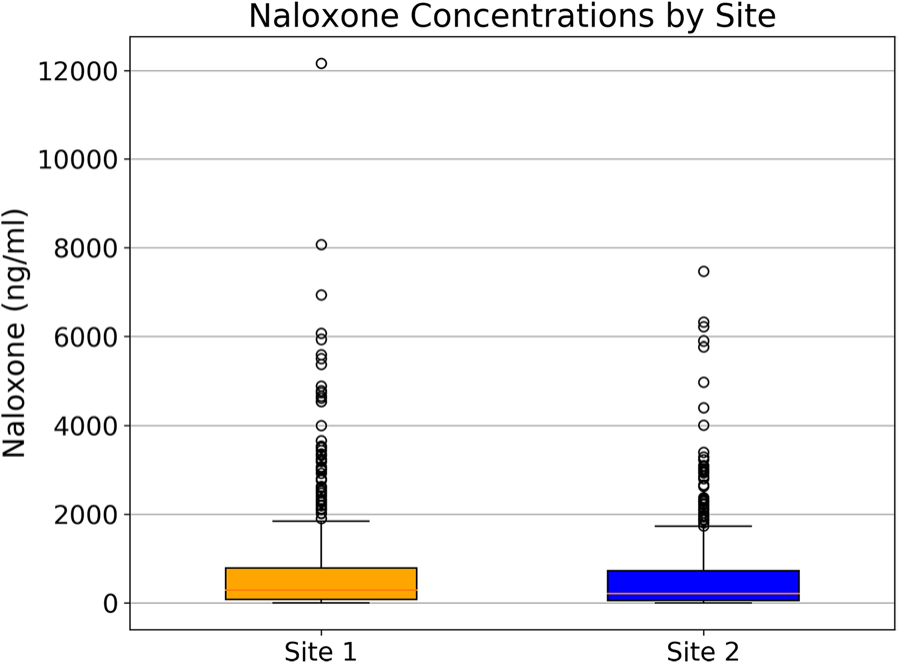
Naloxone concentrations by Site: A box-and-whisker plot of the distribution of naloxone levels (ng/ml) at Sites 1 and 2 is presented. Each box depicts the 25th and 75th percentiles with the median depicted by an orange horizontal line. The whiskers extend above the 75th percentile by 1.5 times the interquartile range for each site. Values outside of the whiskers are marked individually

**Fig. 2:**
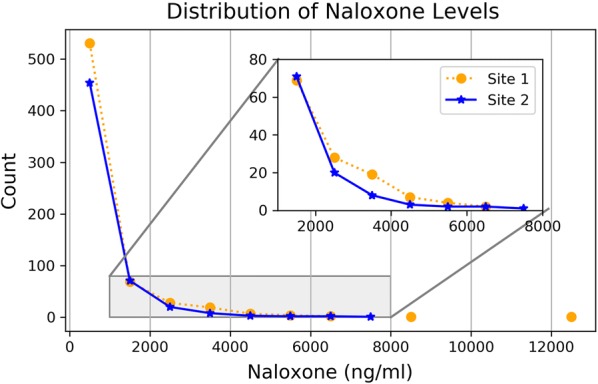
Distribution of naloxone levels: a line histogram of naloxone concentrations (ng/ml) vs. the occurrence of values (count) is presented. Values are binned by 1000 s. Site 1 (dotted, orange line) and Site 2 (solid, blue line) are compared. An inset focused on a narrower range of values

An analysis of naloxone levels across the two sites was performed to assess if any differences were detectable in this marker. The average naloxone level for patients at Site 1 was 686.8 ng/ml over the 3-month period in comparison to 570.9 ng/ml at Site 2 (see Fig. 1; box and whisker plot; two-sample Student’s T-Test p < 0.05). The proportion of extreme naloxone levels, defined as > 2000 ng/ml, was higher at Site 1 (9.3%) in contrast to Site 2 (6.4%). Further investigation into the differences between these two sites demonstrated a difference across naloxone levels (Fig. 2).

For comparison, we also evaluated the N:B ratios in the high naloxone cases (> 2000 ng/ml) in comparison to those below 2000 ng/ml. We found the mean N:B ratio in the elevated naloxone levels was 2.6 in comparison to the mean N:B ratio of 3.9 in cases with naloxone levels below 2000 ng/ml.

## Discussion and conclusions

Naloxone is extensively metabolized by first-pass metabolism by glucuronidation, N-dealklyation and reduction (Suboxone, package insert). Since naloxone demonstrates low bioavailability through oral or sublingual administration, significantly elevated levels of naloxone are not considered to be routinely identified in urine specimens [14]. A limited number of studies have begun to evaluate naloxone levels in urine [13, 14] and have demonstrated that detectable naloxone levels may present in urine after oral/sublingual administration. For example, in one study, naloxone ranged from 5 to 1700 ng/ml (n = 40 patients, [11]).

In this study, naloxone ranged from 0 to 12,161 ng/ml (n = 1223 samples among 275 unique patients) with 8.0% of samples demonstrating extreme values of naloxone (> 2000 ng/ml). To our knowledge, this is the first study to report naloxone levels as high as 12,161 ng/ml; this may be in part due to reporting limits in the upper range in many laboratories [13]. This study suggests, however, that examination of these upper ranges (> 1000 ng/ml) may provide important insights into clinical care.

A wide range of naloxone levels are seen even at lower naloxone concentrations. This may be related to pharmacokinetic differences in how naloxone is absorbed, distributed, metabolized, and excreted [14]. Individual variability in genetics, nutrition, concurrent medications, and/or the presence of hepatic impairment are among the factors that may alter the urinary levels of naloxone [14].

In this study, we examined differences in naloxone concentrations across two practice settings that shared staffing, provider care, policies and protocol. The only known differences between the two practices were the location of the office and a reported higher rate of witnessed attempts to tamper at Site 1 in contrast to Site 2. The higher rate of tampering at Site 1 was further corroborated by the identification of a doubling of flagged specimen validity tests at Site 1 in comparison to Site 2.

While the median values between the two sites were somewhat similar (Site 1: 290 ng/ml; Site 2: 207 ng/ml), we found a higher percentage of samples with extreme values of naloxone, as defined by greater than 2000 ng/ml at the site of possible tampering (Site 1; 9.3%) in contrast to the site with no significant report of tampering (Site 2; 6.4%).

It was not surprising that the median naloxone values were similar between the two practices because the two provider locations were similar in nearly every respect: a single provider with shared staffing, policies and protocol and prescribing practices. The significant difference between the two sites was instead in the percent of specimens with naloxone values > 2000 ng/ml, corroborating the clinical impression that tampering, and potentially the practice of spiking samples into urine to simulate treatment adherence, was more common in Site 1.

While differences were identified between Site 1 and Site 2, we cannot definitively conclude these differences can be attributed to increased rates of “spiking” of urine specimens. There remain several mechanisms by which naloxone may exceed what are typically lower levels in urine, including recent Narcan administration, intravenous naloxone administration or pharmacokinetic alterations in gastrointestinal absorption, metabolism, or distribution [14, 15]. A more systematic evaluation correlating individual naloxone levels with associated buprenorphine levels will be necessary to more fully identify whether naloxone is a reasonable marker for the practice of spiking of urine specimens. However, the association to the clinically-identified observations of tampering attempts between the two sites is supportive of the potential use of naloxone in identifying treatment adherence.

Testing for naloxone in urine is a readily available option in the clinical laboratory. While additional examination is required, this study supports that naloxone may serve as a reasonable flag to identify potential spiking of buprenorphine–naloxone combination product into urine. This is further substantiated by the lower mean N:B ratio in elevated (> 2000 ng/ml) naloxone cases. This study, to our knowledge, represents the largest collection of cases with demonstrably high naloxone levels. Further, this report demonstrates cases with significantly higher than seen previously for naloxone.

## Data Availability

The datasets generated and/or analyzed during the current study are not publicly available due to the need to support the privacy of patients and providers but are available in an aggregated, de-identified from the corresponding author on reasonable request.

## Abbreviations

MOUD: medications for opioid use disorder
OUD: opioid use disorder
LC–MS/MS: liquid chromatography–tandem mass spectrometry
